# SIRT4 Expression Ameliorates the Detrimental Effect of Heat Stress via AMPK/mTOR Signaling Pathway in BMECs

**DOI:** 10.3390/ijms232113307

**Published:** 2022-11-01

**Authors:** Qiang Ding, Yue Wang, Shu-Wen Xia, Fang Zhao, Ji-Feng Zhong, Hui-Li Wang, Kun-Lin Chen

**Affiliations:** Key Laboratory of Crop and Animal Integrated Farming/Institute of Animal Science, Jiangsu Academy of Agricultural Sciences, Ministry of Agriculture and Rural Affairs, Nanjing 210014, China

**Keywords:** SIRT4, heat stress, lactation, mitochondrial function, AMPK/mTOR signaling pathway

## Abstract

Sirtuin 4 (SIRT4), a member of the SIRT family, has been reported to be a key factor involved in antioxidant defense in mitochondria. This study aimed to explore the potential molecular mechanism via which SIRT4 regulates heat stress-induced oxidative stress and lactoprotein synthesis in bovine mammary epithelial cells (BMECs). Our results showed that SIRT4 was significantly decreased in heat stressed mammary tissue. Depletion of SIRT4 in BMECs induced the generation of ROS, which, as exhibited by the decreased activity of antioxidant enzymes, changed mitochondrial morphology through mediating protein and mRNA levels related to mitochondrial fission and fusion. Moreover, we found that depletion of SIRT4 or stress conditions inhibited the expression of milk proteins, as well as lipid and glucose synthesis-related genes, and activated the AMPK/mTOR signaling pathway. Increased SIRT4 expression was found to have the opposite effect. However, blocking the AMPK/mTOR signaling pathway could inhibit the regulatory function of SIRT4 in milk synthesis-related gene expression. In summary, our results suggest that SIRT4 may play critical roles in maintaining mammary gland function by regulating the AMPK/mTOR signaling pathway in dairy cows, indicating that SIRT4 may be a potential molecular target for curing heat stress-induced BMEC injury and low milk production in dairy cows.

## 1. Introduction

Heat stress is an important factor affecting milk production in dairy cows in summer. Dairy cows are very sensitive to high temperatures and humidity, as these factors disrupt their internal heat homeostasis, resulting in a heat stress response [1], which in turn causes decreased feed intake, increased respiratory frequency and rectal temperature, and endocrine disorders [2,3]. Moreover, heat stress usually reduces the number of mammary acini and induces mastitis in lactating dairy cows [4]. Therefore, the normal function of the bovine mammary gland is one of the critical factors that determine milk production. Dairy cows often suffer from abnormal mammary gland growth and development under heat stress conditions [5]. Various studies have shown that heat stress disrupts cytosolic Ca^2+^ homeostasis and induces oxidative stress. Excessive reactive oxygen species (ROS) production usually induces mitochondrial dysfunction, leading to autophagy and apoptosis in bovine mammary epithelial cells (BMECs). In addition, heat stress can change the expression of key lactation genes, thus affecting milk production [6,7,8]. Therefore, finding a strategy to prevent heat stress-induced injury and changes in milk synthesis-related genes in BMECs is necessary to attenuate heat stress-induced low milk production in dairy cows.

Sirtuin 4 (SIRT4), a member of the NAD^+^-dependent deacetylase family, is distributed in the mitochondria [9], where it can be identified through its N-terminal mitochondrial signal sequence and plays an important role in mitochondrial adenosine 5′-triphosphate (ATP) homeostasis, redox, and glutamate metabolism [10]. Studies have shown that SIRT4 can facilitate the coupling of mitochondrial respiration with oxidative phosphorylation, resulting in efficient ATP generation. SIRT4 depletion activates mitochondrial biogenesis and fatty-acid oxidation by mediating the AMPK–PGC1α pathway [11]. Moreover, SIRT4 can promote mitochondrial fusion by interacting with optic atrophy 1 (OPA1) to counteract stress-induced mitochondrial fission and mitophagy. In addition, SIRT4 regulates malondialdehyde CoA content through acetylated mitochondrial malondialdehyde CoA decarboxylase activity, thus regulating fatty-acid metabolism [11,12,13]. In mouse adipose and muscle cells, knockdown of SIRT4 promotes fatty-acid oxidation and reduces fat production, while overexpression of SIRT4 reverses this phenomenon [14]. In addition to regulating enzyme activity, SIRT4 regulates lipid metabolism-related genes by mediating the activation of transcription factors. SIRT4 depletion induces AMP-activated protein kinase (AMPK) activation and SIRT1 upregulation, which are involved in the oxidative metabolism of fatty acids by increasing the transcription of PPARα-mediated genes [15].

AMPK acts as a major regulator of cellular energy metabolism by activating ATP production pathways and blocking ATP consumption [16]. When AMP or ADP increases owing to decreased ATP content in cells, AMPK inhibits anabolic pathways and regulates catabolic pathways to produce more ATP [17,18]. AMPK is activated by hypoxia, glucose deficiency, ischemia, and oxidative phosphorylation. A reduced ATP/AMP ratio activates AMPK, which is regulated by phosphorylation and dephosphorylation at the translational level [19]. The mammalian target of rapamycin (mTOR) protein is a member of the phosphatidylinositol-associated kinase protein family, which regulates cell growth, proliferation, differentiation, and lactoprotein synthesis. It also plays an important role in gene transcription and protein synthesis [20]. mTORC1 is a cell perception center that connects environmental signals and metabolic processes to maintain cell homeostasis. Studies have shown that mTORC1 inactivation under stress conditions stimulates the formation of pre-autophagic complexes [21,22,23]. Adrenomedullin may regulate autophagy responses through the AMPK/mTOR signaling pathway [24]. In addition, AgNPs induced oxidative stress and mitochondrial damage-mediated autophagy in mouse mammary epithelial HC11 cells through the Akt/AMPK/mTOR pathway [25].

Given the potential function of SIRT4 in mitochondria and the probable relationship between SIRT4 and AMPK, we used a high-temperature-induced BMECs heat stress model to investigate whether SIRT4 is involved in heat stress-induced BMECs injury and the reduction of milk production in dairy cows.

## 2. Results

### 2.1. SIRT4 Is Downregulated in Heat-Stressed Bovine Mammary Tissue Samples

**Figure 1:**
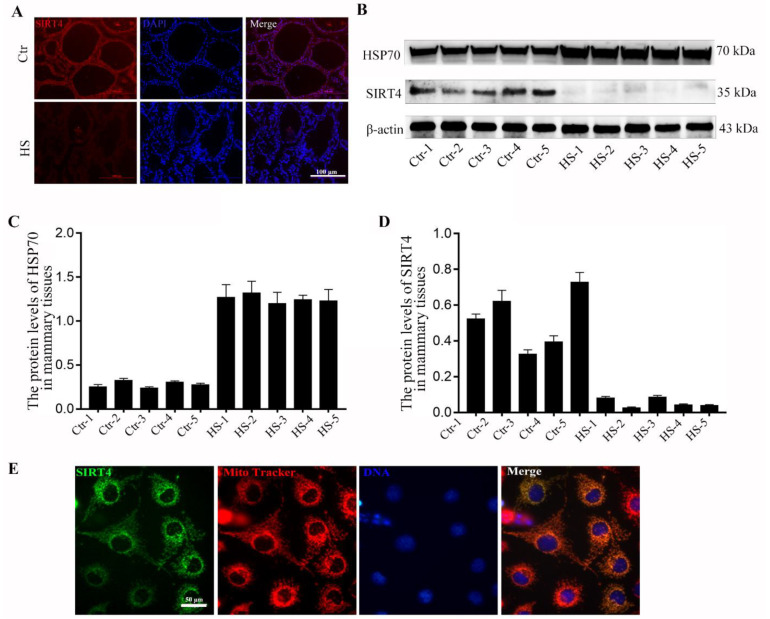
SIRT4 expression and localization in dairy cow mammary tissue. (**A**) The localization of SIRT4 in mammary tissue in control and heat stress-treated group. (**B**–**D**) The protein and mRNA levels of SIRT4 and HSP70 in mammary tissues were examined by Western blot and qRT-PCR after heat stress treatment. (**E**) SIRT4 was colocalized with mitochondria in BMECs.

To understand whether SIRT4 is involved in heat stress-induced mammary gland injury, we first determined the expression levels of SIRT4 in non-heat-stressed and heat-stressed mammary tissues. As shown in Figure 1A, the fluorescence signal of SIRT4 was obviously decreased compared to that in the control group. We used Western blotting to examine the protein level of SIRT4 in bovine mammary tissues. The increased HSP70 expression in heat stress-treated mammary tissue further demonstrated that the cows were under heat stress conditions during the collection period. As expected, SIRT4 expression was significantly downregulated in the five heat-stressed bovine mammary tissues compared to that in the control group (Figure 1B–D). To further explore the functional mechanism of SIRT4 in heat stress-induced mammary gland damage, we used bovine mammary epithelial cells (BMECs) to study the regulatory mechanism of SIRT4 in BMECs. By transfecting the SIRT4 plasmid into BMECs, we found that SIRT4 was colocalized in mitochondria (Figure 1E), indicating that SIRT4 might play critical roles in regulating mitochondrial function.

### 2.2. SIRT4 Involved in Heat Stress-Induced Oxidative Stress in BMECs

**Figure 2:**
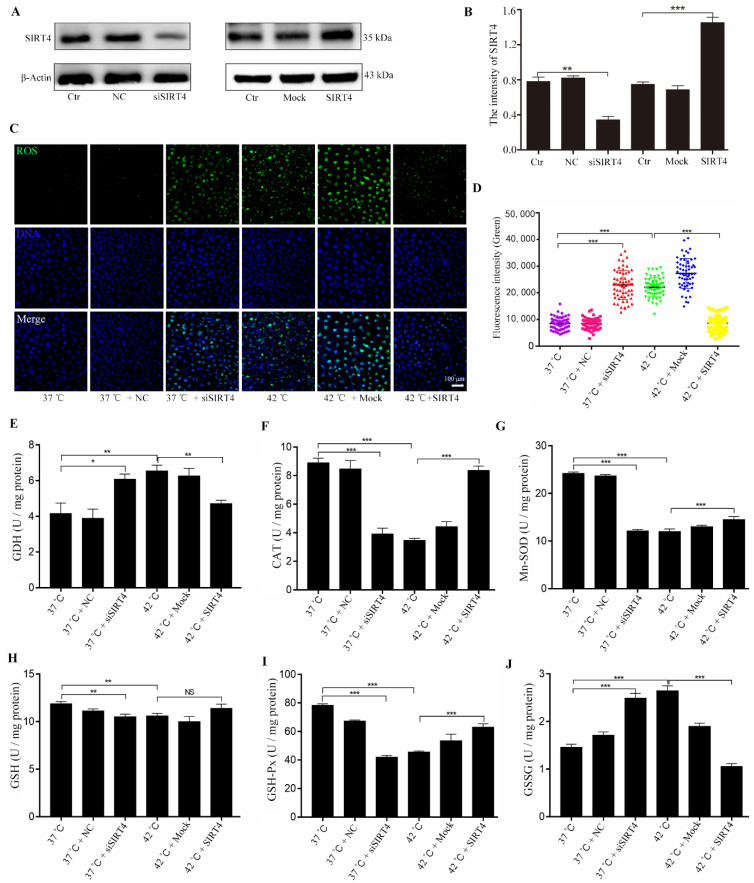
Expression of SIRT4 alleviates oxidative stress of BMECs induced by heat stress. (**A**,**B**) Western blotting was used to detect expression and knockdown efficiency of SIRT4 in BMECs. (**C**,**D**) Expression of SIRT4 alleviated heat stress-induced ROS in BMECs. (**E**–**J**) The activities of CAT, Mn-SOD, and GSH-Px enzymes and the contents of GDH, GSH, and GSSG were detected after expression SIRT4 under heat stress treatment in BMECs. NC: negative control; Mock: expression of control vector. These results are presented as the mean ± SEM from three independent experiments. * *p* < 0.05, ** *p* < 0.01, *** *p* < 0.001.

To investigate the biological function of SIRT4 in the heat stress-induced oxidative stress response, small interfering RNAs targeting SIRT4 were designed and transfected into BMECs. Results showed that SIRT4 levels were significantly reduced after siRNA transfection (Figure 2A). The SIRT4–N13PT–PSVM plasmid was transfected into BMECs for SIRT4 expression. As shown in Figure 2B, SIRT4 protein levels were significantly increased after SIRT4–N13PT–PSVM transfection compared to those in the control group. Our results showed that ROS levels significantly increased after SIRT4 depletion and heat stress treatment in BMECs. Most importantly, the activity of oxidative stress associated enzymes, including CAT, Mn-SOD, GSH, and GSH-Px, was significantly decreased after heat stress treatment and SIRT4 depletion. Consistently, heat stress treatment and SIRT4 depletion increased antioxidant enzyme activity, such as GDH and GSSG. However, SIRT4 expression significantly alleviated heat stress and SIRT4 depletion-induced oxidative stress in BMECs, as shown by decreased oxidative stress enzyme activity and increased antioxidant enzyme activity in BMECs (Figure 2E–J).

### 2.3. SIRT4 Attenuates Heat Stress-Induced Mitochondrial Morphology Defects in BMECs

**Figure 3:**
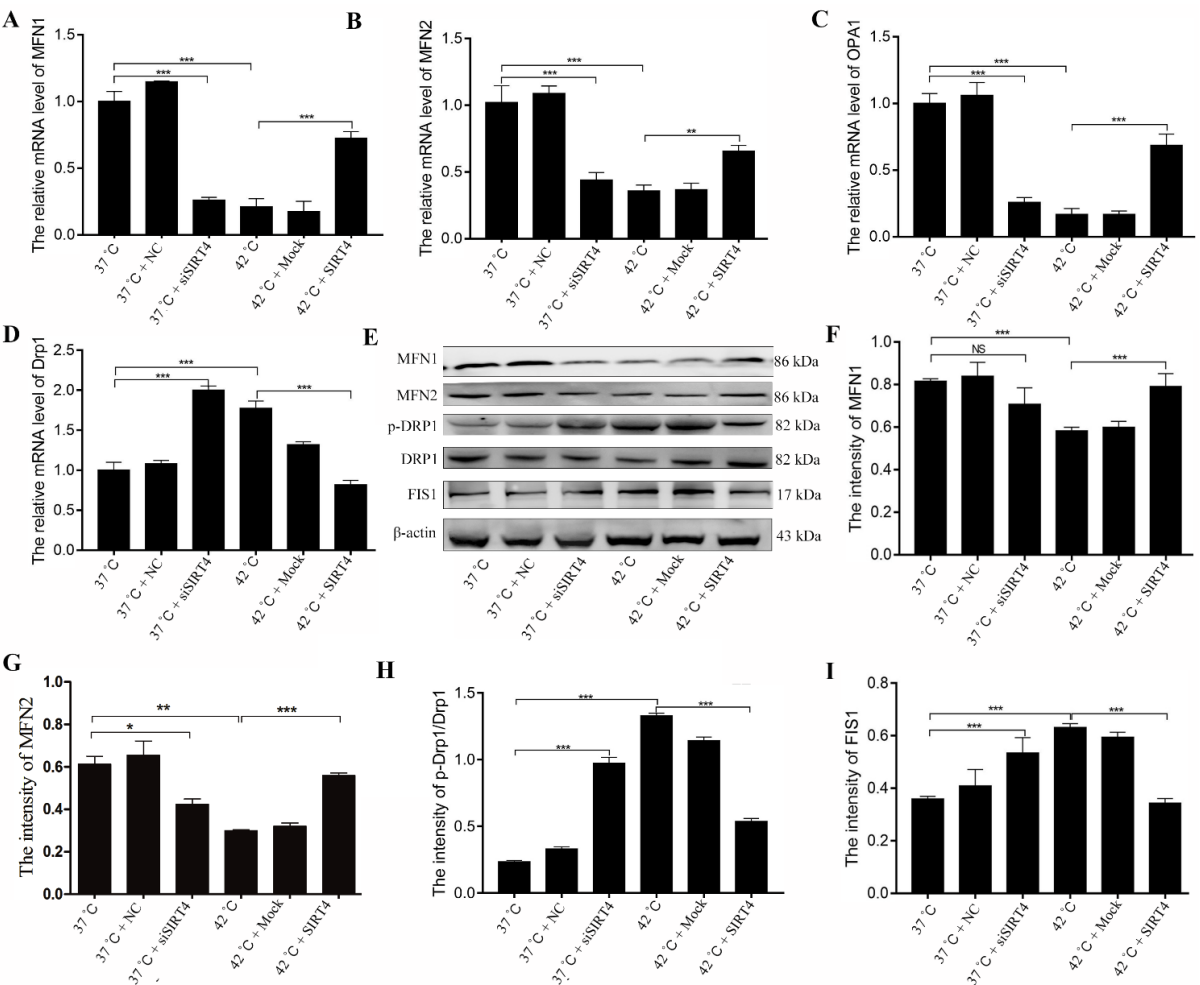
SIRT4 inhibited mitochondrial division and promoted its fusion induced by heat stress. (**A**–**D**) The mRNA levels of *MFN1*, *MFN2*, *OPA1*, and *DRP1* in BMECs were determined by RT-qPCR after expression SIRT4 under heat stress treatment. (**E**–**I**) The protein levels of MFN1, MFN2, p-DRP1, DRP1, and FIS1 were examined in BMECs after expression of SIRT4 under heat stress treatment by Western blotting. NC: negative control; Mock: expression of control vector. These results are presented as the mean ± SEM from three independent experiments. * *p* < 0.05, ** *p* < 0.01, *** *p* < 0.001.

To further explore the potential mechanism of SIRT4 in heat stress-induced oxidative stress, we determined the mRNA and protein levels that regulate mitochondrial fission and fusion processes using quantitative reverse transcription PCR (RT-qPCR) and Western blotting. RT-qPCR results showed that both SIRT4 depletion and heat stress treatment significantly reduced the mitochondrial fusion-related genes *MFN1/2* and *OPA1* (Figure 3A–C). Meanwhile, mRNA levels of *Drp1* and *Fis1*, which are involved in mitochondrial fission, significantly increased after diminishing SIRT4 expression or heat stress treatment (Figure 3D). Consistently, Western blotting results showed that MFN1 and MFN2 levels were significantly decreased under heat stress conditions or SIRT4 depletion (Figure 3E–G). Meanwhile, Drp1 and Fis1 phosphorylation increased after SIRT4 depletion or heat stress treatment, indicating an imbalance in mitochondrial fission and fusion processes (Figure 3E,H,I). However, SIRT4 expression significantly attenuated heat stress-induced aberrant mRNA and protein levels that are involved in mitochondrial fission and fusion (Figure 3E–I).

### 2.4. SIRT4 Activates AMPK/mTOR Signaling Pathway to Inhibit Heat Stress-Induced BMECs Damage

**Figure 4:**
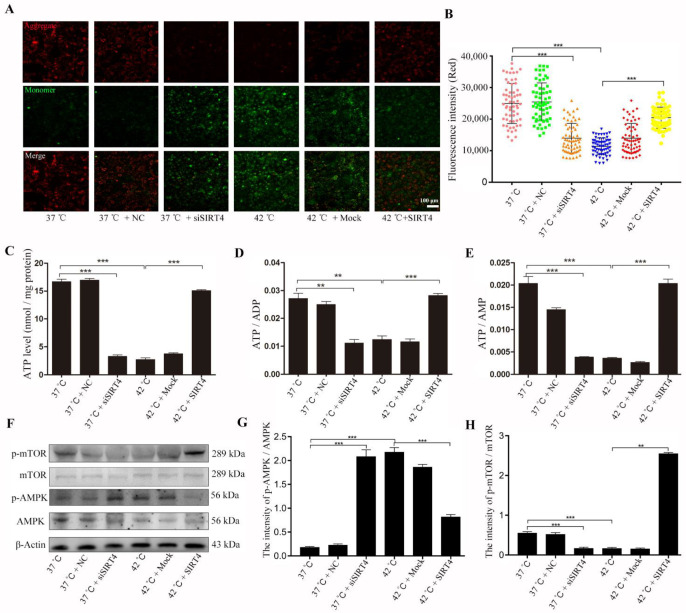
SIRT4 protects BMECs from heat stress-induced cell damage through the AMPK/mTOR signaling pathway. (**A**,**B**) Expression of SIRT4 alleviated heat stress-induced decrease in mitochondrial membrane potential in BMECs. (**C**–**E**) The aberrant contents of ATP, ADP, and AMP were restored after expression of SIRT4 in BMECs. (**F**–**H**) AMPK/mTOR signaling pathway involved in the protective effect of SIRT4 in heat stress-induced damage of BMECs. NC: negative control; Mock: expression of control vector. These results are presented as the mean ± SEM from three independent experiments. ** *p* < 0.01, *** *p* < 0.001.

Given that the destruction of mitochondrial morphology is usually associated with mitochondrial dysfunction, we performed JC-1 staining to examine mitochondrial membrane potential, which is a critical index for evaluating mitochondrial function. The results showed that SIRT4 depletion caused the formation JC1 monomers (green), which was accompanied by the reduced JC1 aggregates (red) in the mitochondrial matrix, indicating a low mitochondrial membrane potential. Consistently, the statistical results showed that the fluorescence intensity of aggregate/monomer was reduced after depletion of SIRT4 or heat stress treatment (Figure 4A,B). Furthermore, ATP, ADP, and AMP levels were also reduced in BMECs, indicating deterioration of mitochondrial function. Nevertheless, increased expression of SIRT4 significantly ameliorates the detrimental effect of heat stress on mitochondrial function, which is exhibited by the increased mitochondrial membrane potential and ATP, ADP, and AMP content in BMECs (Figure 4C–E). We found that the AMPK/mTOR signaling pathway was activated after diminishing SIRT4 or heat stress treatment, which was accompanied by increased AMPK and mTOR phosphorylation levels. In contrast, SIRT4 expression inhibited the activation of the AMPK/mTOR signaling pathway caused by heat stress in BMECs (Figure 4F–H).

### 2.5. SIRT4 Alleviates Heat Stress-Induced Reduction of Lactation Synthesis-Related Genes in BMECs

**Figure 5:**
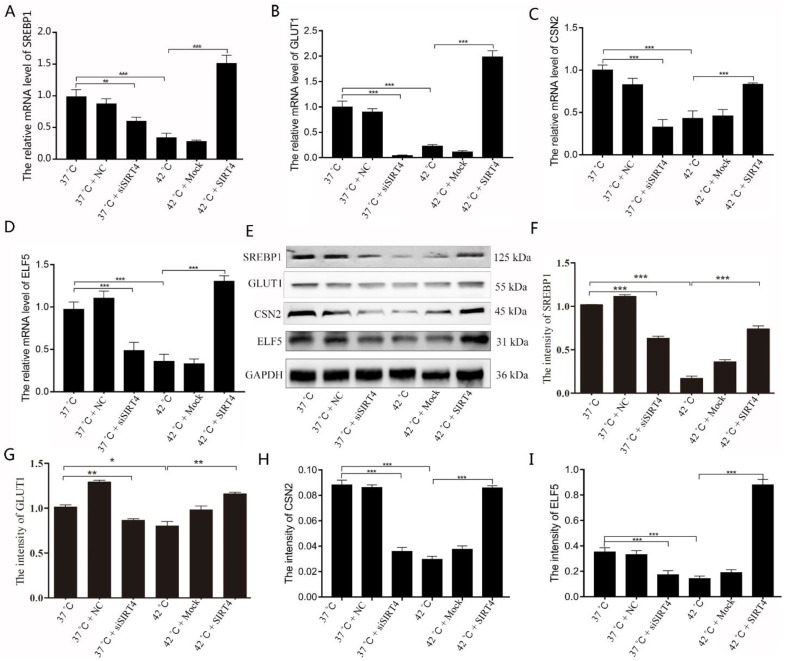
SIRT4 alleviated heat stress-reduced milk synthesis in BMECs. (**A**–**D**) The mRNA expression levels of milk proteins, as well as lipid and glucose synthesis-related genes (*SREBP1*, *GLUT1*, *CSN2*, and *ELF5*), in BMECs were detected by RT-qPCR after expression of SIRT4. (**E**–**I**) Western blotting was used to detect the protein levels of SREBP1, GLUT1, CSN2, and ELF5 in SIRT4-expressed BMECs under heat stress conditions. NC: negative control; Mock: expression of control vector. These results are presented as the mean ± SEM from three independent experiments. * *p* < 0.05, ** *p* < 0.01, *** *p* < 0.001.

To better understand whether the SIRT4-mediated AMPK/mTOR signaling pathway is involved in milk fat and lactation, we explored the mRNA and protein levels of the genes and proteins involved in lactation. Our results showed that mRNA levels of *SREBP1*, *GLUT1*, *CSN2*, and *ELF5* were remarkably reduced after SIRT4 depletion or heat stress treatment (Figure 5A–D). Consistently, Western blotting results showed that the SREBP1, GLUT1, CSN2, and ELF5 levels were also decreased under heat stress conditions or depletion of SIRT4. However, abnormal gene expression or protein levels associated with lactation were restored after SIRT4 expression in BMECs (Figure 5E–I).

### 2.6. Inhibition of AMPK Blocks the Positive Function of SIRT4 in Milk Synthesis in BMECs

**Figure 6:**
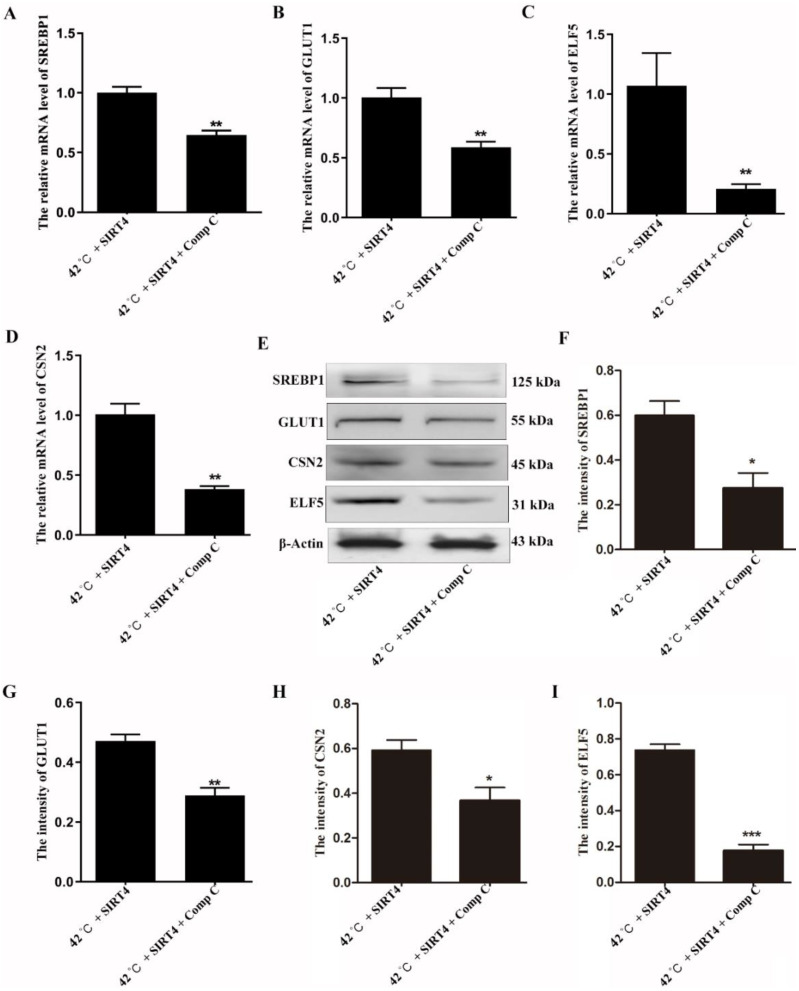
Inhibition of AMPK blocks the positive function of SIRT4 in milk synthesis in BMECs. (**A**–**D**) Compound C treatment inhibited the mRNA expression levels of *SREBP1*, *GLUT1*, *CSN2*, and *ELF5* in SIRT4-expressed BMECs under heat stress conditions. (**E**–**I**) Western blotting results showed that the protein levels of SREBP1, GLUT1, CSN2, and ELF5 were reduced after inhibition of AMPK in SIRT4-expressed BMECs. NC: negative control; Mock: expression of control vector. These results are presented as the mean ± SEM from three independent experiments. * *p* < 0.05, ** *p* < 0.01, *** *p* < 0.001.

To determine the function of the AMPK/mTOR signaling pathway in SIRT4-regulated lactation, the AMPK inhibitor compound C was used to explore whether it could block the regulatory functions of SIRT4 in milk synthesis. Results showed that compound C treatment significantly reduced milk synthesis-associated genes of mRNA levels after SIRT4 expression under heat stress conditions, including *SREBP1*, *GLUT1*, *CSN2*, and *ELF5* (Figure 6A–D). Consistently, Western blot results further demonstrated that AMPK is involved in SIRT4-regulated milk synthesis by mediating SREBP1, GLUT1, CSN2, and ELF5 expression (Figure 6E–I).

## 3. Discussion

Heat stress is one of the most serious factors in reducing the lactation performance of dairy cows in summer. It has been demonstrated that heat stress can induce oxidative stress and apoptosis in the mammary gland of dairy cows. Moreover, BMECs are sensitive to heat stress, which results in decreased milk synthesis and secretion. Therefore, maintaining the normal function of BMECs is a prerequisite for avoiding heat stress-induced reduction of lactation in dairy cows. SIRT4 has a highly conserved nicotinamide adenine dinucleotide (NAD^+^)-dependent deacetylase, which is involved in the regulation of lifespan, aging, and metabolism [26]. Unlike other members of the family, SIRT4 has no NAD^+^-dependent deacetylase activity and instead has ADP-ribosyltransferase activity, which converts glutamate to α-ketoglutarate in the mitochondria [27]. It has also been found to be involved in the regulation of ROS generation in mitochondria. SIRT4 expression can prevent apoptosis by increasing mitochondrial membrane potential and reducing ROS production in podocytes. In addition, it plays a protective role in vascular endothelial cell injury by inhibiting vascular endothelial cell apoptosis induced by oxidized low-density lipoprotein [28]. Consistently, we found that reduced SIRT4 expression was involved in heat stress-induced oxidative stress. The expression of SIRT4 can alleviate heat stress-induced ROS levels by increasing the activities of antioxidant enzymes.

Mitochondria are the major sources of ROS, and mitochondrial dysfunction often causes excessive ROS production, resulting in oxidative stress and apoptosis [29,30,31]. Previous studies have shown that mitochondrial dynamics is an essential quality control method involved in mitochondrial fission and fusion [32]. MFN1/2 and OPA1 contribute to mitochondrial fusion [33,34], and Drp1 and Fis1 are responsible for mitochondrial fission [35,36,37]. Mitochondrial fusion allows for the swapping of mitochondrial contents and the repair of cellular damage. SIRT4 has been shown to interact with OPA1 to promote mitochondrial fusion, which prevents mitochondrial autophagy and regulates mitochondrial mass. Our results showed that SIRT4 expression promotes the expression of mitochondrial fusion-related genes *MFN1/2* and *OPA1* under heat stress conditions; however, the mechanism via which SIRT4 affects mitochondrial fusion-related gene expression is unknown. In addition, mitochondrial fission is closely associated with apoptosis, which is regulated by the outer mitochondrial membrane. Drp1 is a key regulator of mitochondrial fission. SIRT4 depletion can induce mitochondrial fission by increasing ERK-regulated phosphorylation of Drp1 [38]. Our previous study showed that heat stress can promote mitochondrial fission by increasing the phosphorylation of Drp1 in BMECs. In the present study, we found that SIRT4 is involved in the aberrant mitochondrial morphology induced by heat stress. Expression of SIRT4 can inhibit the heat stress-induced increase in Drp1 phosphorylation and Fis1 expression; however, it is necessary to further explore the regulatory mechanism between SIRT4 and mitochondrial fission-related genes. This indicates that SIRT4 can reduce heat stress-induced oxidative stress by improving mitochondrial function.

The key role of oxidative stress induced by heat stress is to induce mitochondrial membrane permeability transition, resulting in an imbalance in membrane potential homeostasis and ATP synthesis stagnation [39,40,41,42]. Decreased cellular ATP synthesis can increase ADP and AMP content, which further activates the AMPK/mTOR signaling pathway [43,44]. AMPK is the central node that coordinates cellular metabolism and specific energy requirements. When cell energy levels are low, AMPK is activated, which further reduces mTOR phosphorylation and autophagy [45]. Previous studies have shown that SIRT4 regulates regulatory T-cell generation and function by inhibiting AMPK signaling. Therefore, we speculate that the regulatory function of SIRT4 in oxidative stress is associated with AMPK activation. Following SIRT4 expression in BMECs, we found that aberrant mitochondrial membrane potential and ATP synthesis caused by heat stress were restored. The AMPK/mTOR signaling pathway was also inhibited. These results indicate that SIRT4 may alleviate mitochondrial function by inhibiting the AMPK/mTOR signaling pathway activation.

SREBP1 is a determinant of milk fat synthesis in the mammary glands of dairy cows and can regulate the expression of genes related to fatty-acid synthesis. Studies have shown that acetic acid regulates SREBP1 through the mTOR pathway and affects milk fat synthesis [46]. In addition, it has been found that prolactin can activate mTOR signaling pathway through the phosphorylation of the PI3K/PKB pathway, thus regulating lactoprotein translation [47]. AMPK is also involved in milk fat and protein synthesis by mediating PGC-1α acetylation [48]. In line with previous work, the present study showed that AMPK inhibition blocked SIRT4 regulation in milk fat and lactoprotein synthesis-related genes in BMECs, indicating that the SIRT4-regulated AMPK/mTOR signaling pathway is involved in heat stress-induced low-quality milk production in dairy cows. As expected, the results indicated that SIRT4 expression alleviated milk fat synthesis inhibition caused by heat stress in BMECs.

## 4. Materials and Methods

### 4.1. Collection of Cow Mammary Tissue Sample

Heat stress was assessed by recording barn temperature and humidity, calculating the temperature and humidity index (THI, THI = 0.81 Td + (0.99 Td − 14.3) RH + 46.3), and measuring rectal temperature and respiratory rate. We collected five normal and five heat stress mammary tissue samples from Chinese Holstein cows in a local slaughterhouse; their age and parity were basically the same, and the basic traits of cattle are shown in Supplementary Table S1. Within 20 min after slaughter, a sample of breast tissue was quickly collected and divided into two parts, one of which was stored in liquid nitrogen until analysis, while the other was fixed in 4% paraformaldehyde for use.

### 4.2. Cell Culture, Plasmid Construction, Transfections, and Heat Stress Treatment

Bovine mammary epithelial cell lines (BMECs) were cultured in DMEM/F-12 Medium (Basal Media, Shanghai, China) with 10% fetal bovine serum (FBS) (Biological Industries, Shanghai, China) under 37 °C in a humidified incubator with 95% air and 5% CO_2_. BMECs were passaged at ~80% confluence every 3–4 days with 0.05% trypsin/EDTA solution.

The Sirt4 fragment was subcloned into the NIPT-PSVM vector (provided by Shao-Xian Cao, Jiangsu Academy of Agricultural Sciences). Then, transfections were performed using Lipofectamine 2000 (Thermo Fisher Scientific, Waltham, MA, USA) according to the manufacturer’s instructions. Transfection efficiency was determined via RT-qPCR and Western blotting. Table S2 lists the SIRT4 small interfering RNA sequence synthesized by GenePharma in Supplementary Table S2.

To establish the heat stress model, the cells were incubated at 42 °C in an incubator for 3 h to mimic the high-temperature environment in summer. After that, the cells were subjected to different experiments.

### 4.3. Immunofluorescence Staining

BMECs were fixed with 4% PFA for 30 min at room temperature. After washing three times with PBS, the cells were permeabilized with 0.5% Triton X-100 for 20 min, and then blocked with 1% BSA for 1 h at room temperature. Samples were incubated with primary antibody (SIRT4 1:100) at 4 °C overnight, and then labeled with secondary antibody for 1 h at room temperature. After washing three times with washing buffer, DNA was stained with DAPI for 10 min. Samples were imaged with fluorescence microscope.

### 4.4. RNA Extraction and RT-qPCR

Total RNA was extracted using the Total RNA Kit I Kit (Omega Bio-Tek, Norcross, GA, USA), followed by reverse transcription using the HiScript II Q RT SuperMix for qPCR (Novizan, Nanjing, China) Kit. Reverse transcription cDNA was used as the template, and a ChamQ SYBR qPCR Master Mix (Low ROX Premixed) (Novizan, Nanjing, China) kit was used to quantitatively detect mRNA expression levels of *MFN1*, *MFN2*, *OPA1*, *DRP1*, *GLUT1*, *CSN2*, and *ELF2* in cells. NA. Expression levels of all genes were normalized to those of endogenous reference gene β-actin, according to an optimized comparative Ct^(2-ΔΔCt)^ value method, where ΔΔ = ΔCt_target_ − ΔCt_β-actin_. Primer sequences are listed in Table S3 of the Supplementary Materials.

### 4.5. Western Blotting

Total protein was extracted using a kit from Sangon Biotech (Shanghai, China) according to the manufacturer’s instructions. Then, the total concentration was measured using an enhanced BCA Protein Assay Kit (CWBIO, Jiangsu, China). After that, the Qestern blot procedures were performed as previously described [9]. Briefly, the protein samples were denatured at 100 °C for 5 min, then separated on 12% sodium dodecyl sulfate–polyacrylamide gel electrophoresis mini gels (GenScript, Shanghai, China), and blotted to PVDF membranes (Millipore, Bedford, MA, USA). Afterward, the blots were incubated with 5% nonfat milk for 1 h and incubated with primary antibody overnight. Subsequently, the blots were washed three times with TBST (10 min/time) before incubation with anti-rabbit IgG, HRP-linked antibody or anti-mouse IgG, HRP-linked antibody for 1 h. After the blots were washed with TBST three times, the enhanced chemiluminescence signal was detected using an ECL chemiluminescence kit and analyzed using image J software (NIH, Bethesda, MD, USA).

The commercially primary antibodies that used in this study were as follows: anti-Mn-SOD [9] (1:3000; Proteintech, Chicago, IL, USA), anti-β-actin (1:4000; Proteintech, Chicago, IL, USA), anti-HSP70 (1:4000; Proteintech, Chicago, IL, USA), anti-MFN1 (1:2000; Abcam, Cambridge, UK), anti-MFN2 (1:2000; Proteintech), anti-FIS1 (1:2000; Proteintech), anti-p-DRP1 (1:2000; Affinity, Changzhou, China), anti-DRP1 (1:2000; Affinity). anti-OPA1 (1:2000; Proteintech), anti-GLUT1 (1:2000; Affinity), anti-CSN2 (1:2000; Clous-Clone Corp, Wuhan, China), anti-ELF5 (1:2000; Affinity), anti-SIRT4 (1:2000; Affinity), anti-SREBP1 (1:2000; Proteintech), anti-AMPK (1:2000; Cell signaling technology), anti-p-AMPK (1:1000; Cell signaling technology), anti-mTOR (1:1000; Affinity), and anti-p-mTOR (1:1000; Affinity).

### 4.6. Mitochondrial Membrane Potential and ROS Assay

A Reactive Oxygen Species Assay Kit (Beyotime, Haimen, China) and MitoProbe JC-1 (Beyotime) were used to detected mitochondrial membrane potential and ROS levels according to the manufacturer’s instructions. Briefly, the cells were incubated with 2′,7′-dichlorodihydrofluorescein diacetate (DCFH-DA) and JC-1 dye at 37 °C in an incubator for 30 min. After washing three times with serum-free medium, the cells were observed using a fluorescence microscope (Nikon, Eclipse Ti-s, Tokyo, Japan).

### 4.7. GDH, CAT, Mn-SOD, GSH, GPx, and GSSG Detection

After different treatment, the cells were washed with PBS and placed in a centrifuge tubule for determining the activity of oxidative enzymes and antioxidative enzymes using GDH, CAT, Mn-SOD, GSH, GPx, and GSSG kit, respectively. These kits were purchased from the Nanjing Jiancheng Bioengineering Institute, and the procedures were performed according to the manufacturer’s instructions. After that the absorbance value was determined at different wavelengths using a spectrophotometer (BioTek Eon, Winooski, VT, USA).

### 4.8. Data Analysis

The data were compared using t-tests for the comparison of two groups with GraphPad Prism software 9 (La Jolla, CA, USA) before graphing, and a *p*-value <0.05 was considered statistically significant. The data are presented as the mean ± standard deviation (SD) from three replicate experiments.

## 5. Conclusions

In summary, the present study demonstrated that SIRT4 has a protective effect on heat stress-induced oxidative stress and mitochondrial dysfunction in BMECs, which further improves the function of BMECs, as well as milk fat and lactoprotein synthesis, by mediating the AMPK/mTOR signaling pathway. These results provide a theoretical basis for developing a solution to the decreased milk production in dairy cows in the summer.

## Data Availability

All data will be made available upon reasonable request by emailing the corresponding author.

## Supplementary Materials

The following supporting information can be downloaded at: https://www.mdpi.com/article/10.3390/ijms232113307/s1.
