# Supplementary material for: SIRT4 Expression Ameliorates the Detrimental Effect of Heat Stress via AMPK/mTOR Signaling Pathway in BMECs
Source: Int J Mol Sci. 2022 Nov 1;23(21):13307. doi: 10.3390/ijms232113307 (PMC9658231; doi:10.3390/ijms232113307)
Supplement: Supplementary file 1 [file ijms-23-13307-s001.zip › ijms-1993256-supplementary.pdf]

Table S1 The Data related to the Holstein cattle sample

Sample	Cowshed temperature(Td) /℃	Cowshed Relative humidity(RH)/%	Temperature and humidity index（THI）	Rectal temperature /℃	Average breathing rate (Times/min)
Ctr-1	11	56	53.30	38.1	47±2.03
Ctr-2	12	53	54.74	38.5	53±2.10
Ctr-3	12	53	54.74	38.5	58±1.89
Ctr-4	11	56	53.30	38.4	52±1.78
Ctr-5	11	56	53.30	38.3	56±2.58
HS-1	28	81	79.85	39.7	106±2.65
HS-2	27	79	77.99	39.8	96±2.00
HS-3	29	79	81.17	40.5	102±2.08
HS-4	29	77	80.89	40.3	108±2.65
HS-5	30	83	83.38	40.2	112±1.53

$THI=0.81Td+(0.99Td-14.3)RH+46.3$

Table S2 The primer sequences for siRNA

Genes	Primer Sequences (5’-3’)
si-SIRT4	F: GGAGAGUUGCUGCCAUUAATT<br>R: UUAAUGGCAGCAACUCUCCTT
Negative control	F: UUCUCCGAACGUGUCACGUTT<br>R: ACGUGACACGUUCGGAGAATT

Table S3 The primer sequences for real-time PCR

Genes	Primer Sequences (5’-3’)
MFN1	F: TGTTTTGGTCGCAAACTCTG<br>R: CTGTCTGCGTACGTCTTCCA
MFN2	F: CGTCAAGAAGGATAAGCGACAC<br>R: TCTGACCACTTCTTACCG
OPA1	F: AAGAGGCACTTCAAGGTCG<br>R: GGGAGGAGGAAGAGCAGA
Drp1	F: TCTCCGAGTCCTTTATTG<br>R: TGCTGTTGAAGTCGCAGGAG
GLUT1	F: GTGCTCCTGGTTCTGTTTCTTCA<br>R: GCCAGAAGCAATCTCATCGAA
CSN2	F: TCTGCCTCTGCTCCAGTCTT<br>R: AGGAGGGGGCATTCACTTT
ELF5	F: GTGGCATCAAGAGTCAAGACTGTC<br>R: CTCAGCTTCTCGTACGTCATCCTG
SREBP1	F: TGGACCAGGCAAGAGAAGAG<br>R: TCTTCCTCCAGCTTGACAGG
β-Actin	F: TCACCAACTGGGACGACA<br>R: GCATACAGGGACAGCACA
